# Arthroscopic Bone Block Cerclage Technique Using a Tricortical Scapular Spine Autograft for Glenoid Reconstruction in Patients With Anterior Shoulder Instability

**DOI:** 10.1016/j.eats.2021.11.004

**Published:** 2022-02-18

**Authors:** Philipp Moroder, Thiele Kathi, Lucca Lacheta, Katrin Karpinski, Alp Paksoy, Doruk Akgün

**Affiliations:** Charité – Universitätsmedizin Berlin, corporate member of Freie Universität Berlin, Humboldt-Universität zu Berlin, and Berlin Institute of Health, Center for Musculoskeletal Surgery, Berlin, Germany

## Abstract

In the treatment of anterior shoulder instability with glenoid bone loss, free bone graft transfers have proven to be a viable anatomic alternative to the commonly performed, nonanatomic Latarjet procedure. Implant-free fixation of the free bone grafts, in particular, has rendered excellent short- and long-term results. However, a drawback remains the source of the graft. We describe an arthroscopic bone block cerclage technique using a tricortical scapular spine autograft, which provides an anatomic arthroscopic glenoid reconstruction with the combined benefit of sparing the subscapularis, metal-free fixation, and intraregional donor site for autograft harvesting.

The extent of glenoid bone loss is one of the most important risk factors leading to recurrent shoulder instability after soft-tissue stabilization procedures.^1^ Thus, in the setting of an extensive bony glenoid defect, bony augmentation techniques such as coracoid transfer, iliac crest autograft, or allografts are recommended.^2^ However, these techniques have drawbacks and complication rates as high as 25%.^3^, ^4^, ^5^ The Latarjet procedure, despite its wide use, violates the normal shoulder anatomy with a permanent split of the subscapularis by the transferred conjoined tendon, with possible structural effects and restriction of the internal rotation.^6^ Furthermore, Latarjet is associated with damage to neurovascular structures during harvest and fixation.^3^^,^^5^^,^^7^ Iliac crest bone graft transfers require an additional surgery site and are associated with donor site morbidity, postoperative pain, immobility, blood loss, hematoma, and nerve injury.^8^, ^9^, ^10^ Further graft options include allogeneic, xenogeneic, and synthetic grafts, with drawbacks including additional costs, potentially lower graft quality and healing potential, higher osteolysis rates, and risk of disease transmission.^11^, ^12^, ^13^ Furthermore, most bony augmentation techniques require application of metal implants for fixation regardless of the graft used, and the presence of metal in implants is associated with hardware failure, residual pain, and graft resorption.^14^, ^15^

The scapular spine has recently been identified as an appropriate source for structural bone autografts in glenoid reconstruction.^11^ Satisfactory results with an autologous scapular spine bone graft were achieved at short-term follow-up in patients with anterior shoulder instability and subcritical bone loss.^16^ The purpose of this Technical Note is to describe an arthroscopic bone block cerclage technique^17^ that uses a tricortical scapular spine autograft for reconstruction of the glenoid in patients with anterior shoulder instability and glenoid bone loss.

## Surgical Technique

The surgical technique is demonstrated in the Video 1.

### Preoperative Assessment and Indications

An elaborate patient history is obtained, and a functional assessment is performed with complete clinical examination to determine the type of instability, functional demand, joint laxity, and possible other pathologies, followed by a detailed radiologic assessment. All patients with multiple episodes of anterior shoulder instability should be evaluated with 3-dimensional computed tomography (CT) to assess humeral and glenoid bone loss. The current technique is indicated in patients with symptomatic anterior shoulder instability and concomitant measured glenoid bone loss >15%.

### Patient Positioning and Diagnostic Arthroscopy

Under general anesthesia and with a routine single-shot antibiotic regimen, the patient is placed in the lateral decubitus position using a vacuum bean bag positioner. The affected arm is fixed with the help of a traction device in 30° of abduction and neutral rotation, with axial and lateral traction applied to distend the shoulder joint and improve intraarticular visualization.

Three regular portals are required for this procedure: standard posterior, anterosuperior, and anteroinferior. We recommend the posterior portal be incised in a horizontal fashion to ease the introduction of the drill guide from posterior. A diagnostic arthroscopy is first performed through the posterior portal to identify concomitant pathologies; however, the anterior glenoid rim and capsulolabral complex can be evaluated best through the anterosuperior portal.

### Capsulolabral Complex Release and Glenoid Preparation

**Fig 1:**
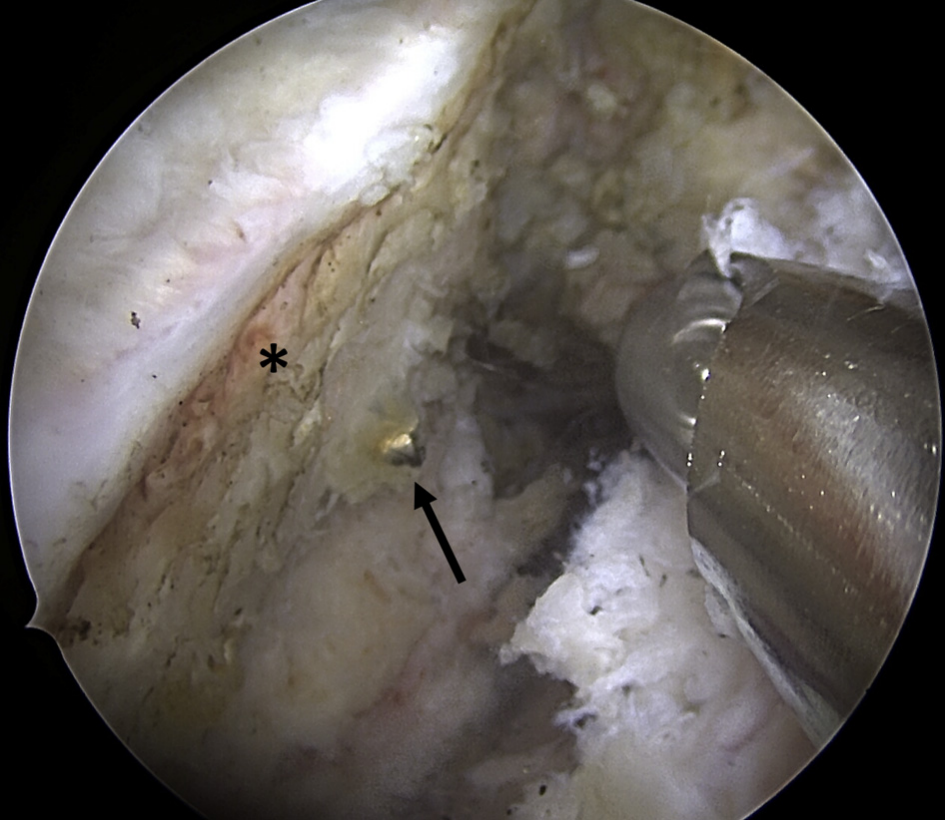
Arthroscopic view of the left shoulder from anterosuperior portal. Patient in lateral decubitus position; after mobilization of the capsulolabral complex and debridement and abrasion of the anterior glenoid defect (∗) with a motorized burr to achieve a plane surface. Note the remaining hardware (arrow) from previous stabilization surgery.

Looking through the anterosuperior portal, the capsulolabral complex is first carefully mobilized using an arthroscopic periosteal elevator. Then the anterior glenoid defect is debrided and abraded with a motorized burr to achieve a flat plane and induce bleeding for improved glenoid bone graft healing potential (Fig 1).

### Glenoid Drilling

**Fig 2:**
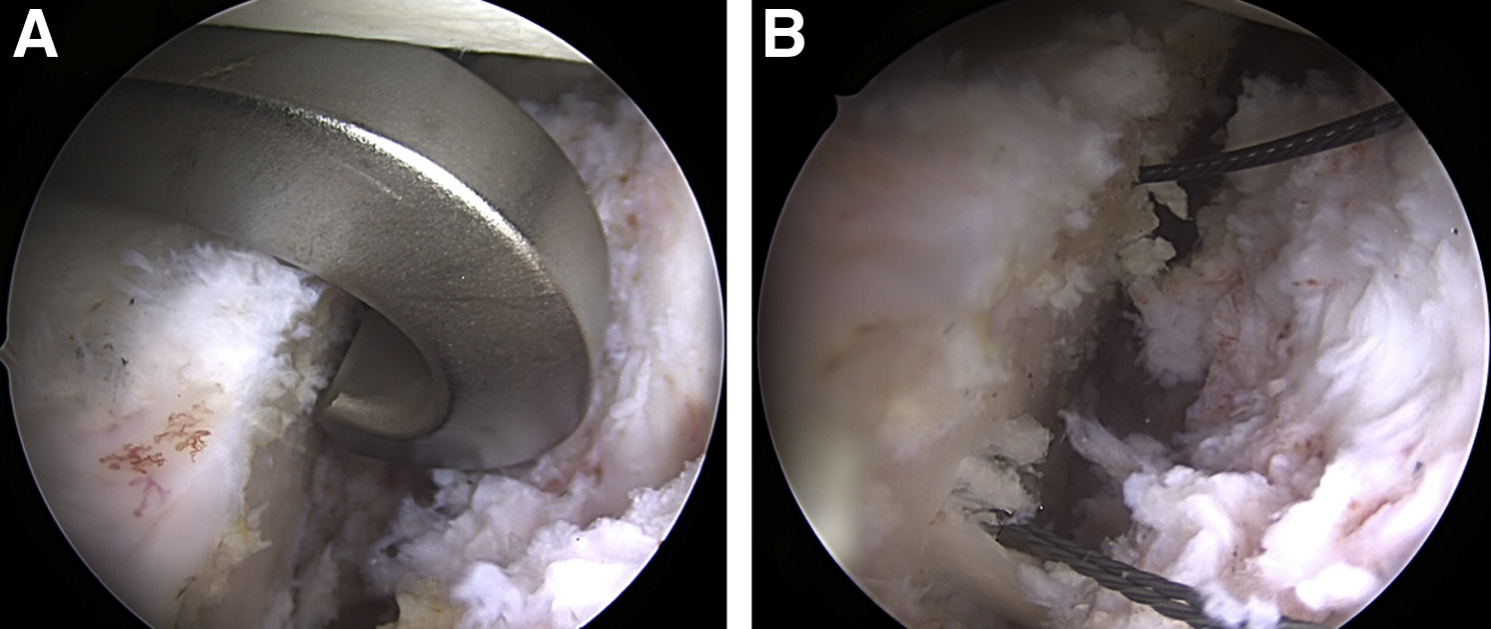
Arthroscopic view of the left shoulder from anterosuperior portal. Patient in lateral decubitus position. (A) Positioning of the drill guide from the posterior portal with the hook position 5 mm deep from the articular surface and parallel to glenoid for correct placement of the drill tunnels. (B) Intraoperative view of nitinol wires shuttled through the drill holes.

**Fig 3:**
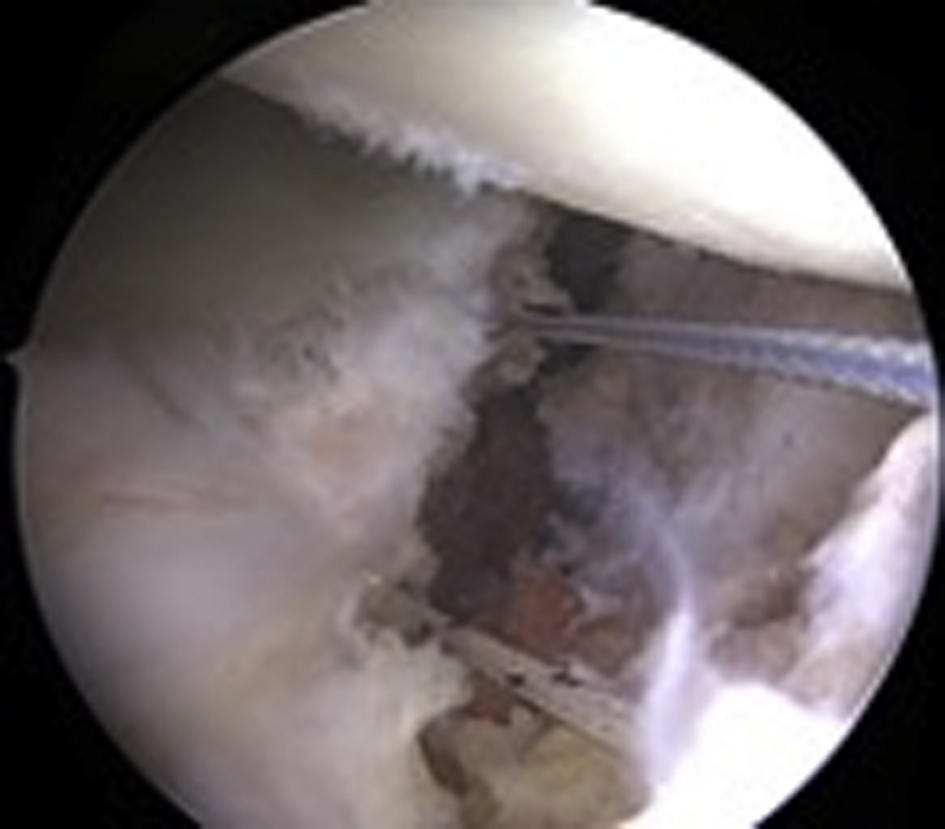
Arthroscopic view of the left shoulder from anterosuperior portal. Patient in lateral decubitus position. Exchange of nitinol wires with FiberLink (blue) and TigerLink (white) sutures for FiberTape-TigerTape Cerclage passage through glenoid and graft tunnels.

An arthroscopic posterior drilling guide (Arthrex, Naples, FL) is introduced into the joint through the posterior portal, and the hook component is placed parallel to the articular surface of the glenoid, as close as possible to the center of the defect and ∼5 mm medial of the cartilage surface (Fig 2A). With the help of the drill guide, two 2.4-mm tunnels are drilled from the posterior scapula neck to the glenoid defect on the anterior scapula neck, parallel to the articular surface and parallel to each other with a 10-mm distance. After removal of the central pins of the cannulated drills, 2 nitinol wires with loops (1 for each tunnel) are passed and retrieved from the anteroinferior portal (Fig 2B). The drill guide, hook, and drills are then removed. Two loop sutures (FiberLink/TigerLink; Arthrex), one with the loop anterior and the other with the loop posterior, are passed through the tunnels using the nitinol wires (Fig 3).

In situ measurement of the anterior glenoid defect is crucial to achieve a perfect fit of the autograft. This is performed by using an arthroscopic measuring probe (220 mm, 60°; Arthrex) from the anteroinferior portal to measure the craniocaudal and mediolateral width of the defect and the exact localization of the drill holes within the defect.

### Scapular Spine Preparation

**Fig 4:**
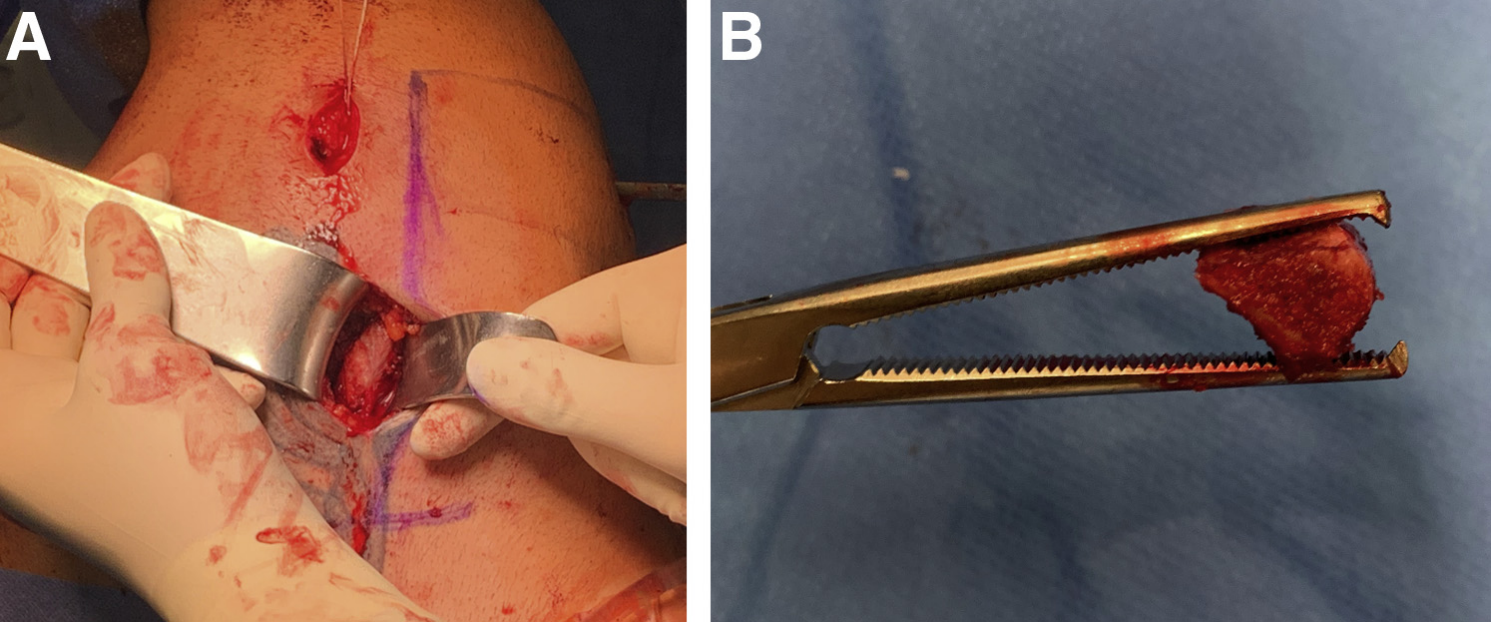
(A) Harvest location 4 to 5 cm lateral to the medial scapular border is selected, which provides the largest cross-sectional graft size while avoiding the acromial base. A horizontal incision is made along the scapular spine, and the posterior deltoid and trapezius fascia are dissected to expose the scapular spine. (B) A tricortical bone graft is harvested from a left shoulder using an oscillating saw. Patient in lateral decubitus position.

A harvest location 4 to 5 cm lateral to the medial scapular border is selected that provides the largest cross-sectional graft size while avoiding the acromial base.^11^ A 5-cm horizontal incision is then made along the scapular spine, and the posterior deltoid and trapezius fascia are dissected to expose the scapular spine (Fig 4A). A tricortical bone graft with a length of 20 mm and a thickness of 8 mm is harvested using an oscillating saw. The width of the graft is predefined by the maximum width of the spina scapulae. The fascia is repaired with resorbable sutures after placing a hemostatic sponge in the harvest area (Fig 4B). After freeing the graft from soft tissue, a slight reshaping can be performed, and two 2.4-mm drill tunnels are made through the graft based on the measurements obtained before.

### Fixation of the Scapular Spine Bone Graft

**Fig 5:**
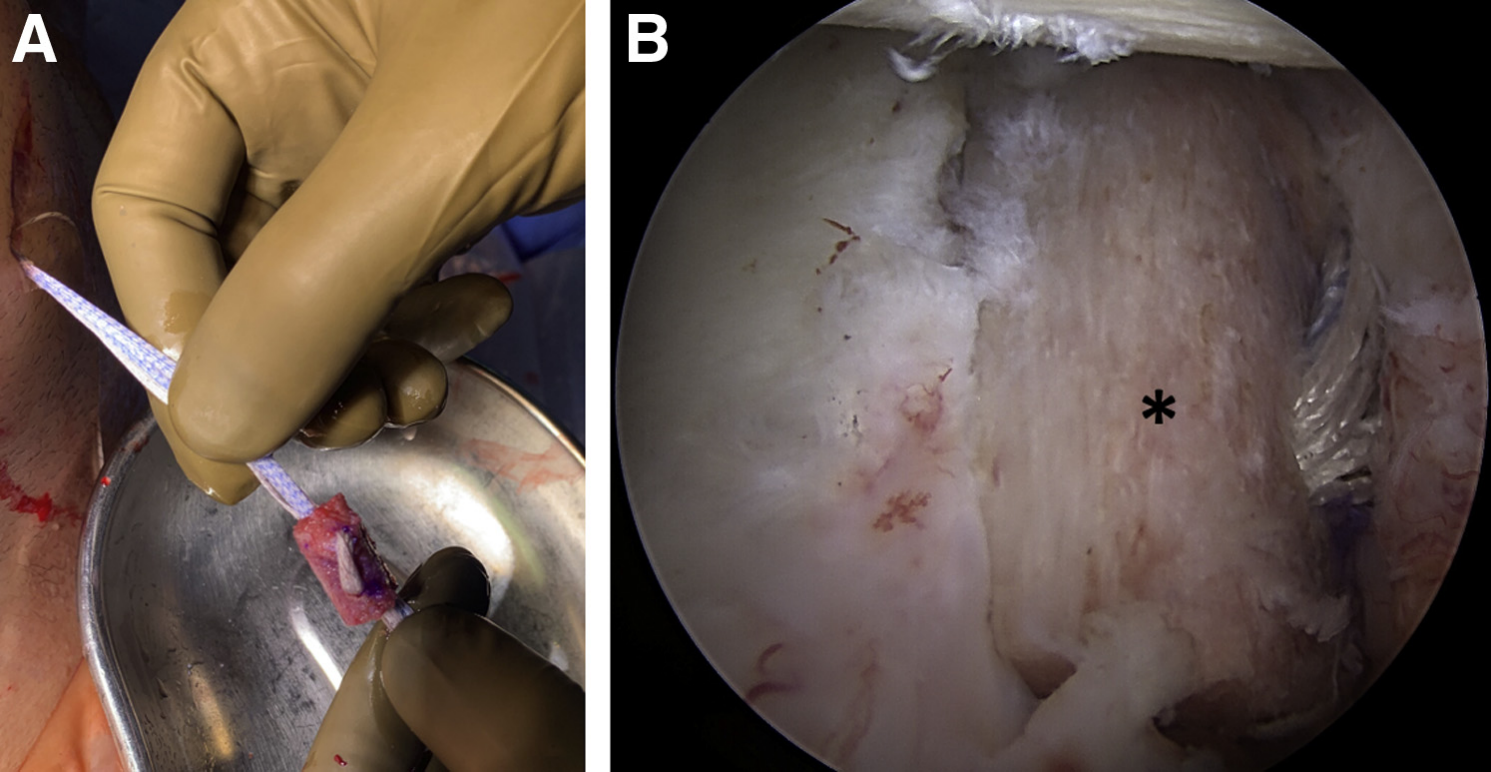
(A) Tricortical scapular spine autograft after FiberTape-TigerTape passage before insertion through the anteroinferior portal and the rotator interval. (B) Arthroscopic view of the left shoulder from anterosuperior portal after positioning of the graft (∗) on the anterior glenoid rim. Patient in lateral decubitus position.

Digital dilation of the anteroinferior portal is performed for better graft passage. Using the inferior FiberLink loop, 2 ultra-high-strength suture tapes (FiberTape Cerclage System; Arthrex) are passed from posterior to the anterior side of the glenoid and retrieved through the anteroinferior portal. The suture tapes are then passed through the inferior tunnel of the autograft from the cancellous side to the cortical side and from the cortical side to the cancellous side through the superior tunnel of the autograft (Fig 5A). The superior TigerLink loop is then loaded with both FiberTape Cerclage sutures to pass them from anterior to the posterior glenoid side. The scapular spine autograft is introduced in the joint through the anteroinferior portal and through the rotator interval by pulling the FiberTape Cerclage sutures while holding the graft with a Kocher clamp.

After insertion and proper positioning of the scapular spine graft (Fig 5B), the FiberTape sutures are interconnected to create a continuous loop. For that purpose, the tail of the FiberTape suture is loaded through the pretied racking hitch knot of the TigerTape and vice versa. To apply sufficient traction on each suture limb in a controlled fashion, a cerclage tensioner set to 60 to 80 N is used. Sutures are then knotted with ≥4 alternating knots, and finally the graft fixation is checked with an arthroscopic probe.

### Capsulolabral Repair

**Fig 6:**
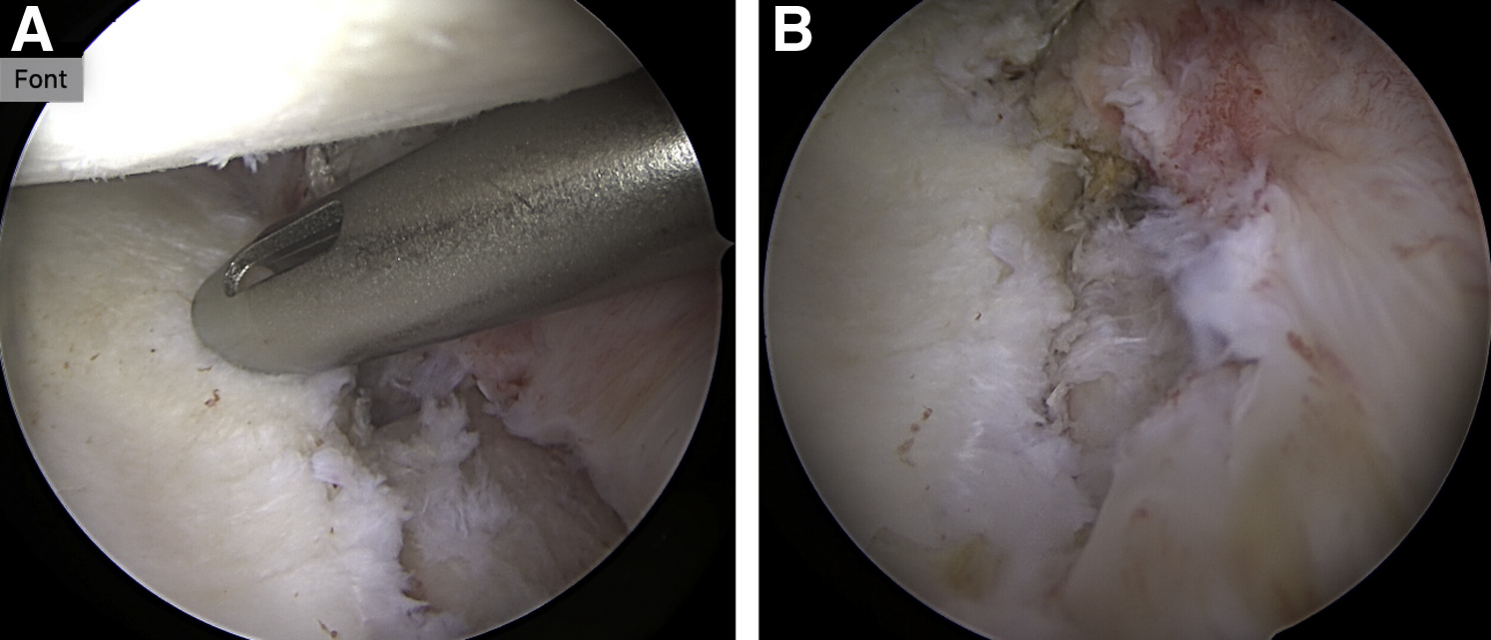
Arthroscopic view of the left shoulder from anterosuperior portal. Patient in lateral decubitus position. Fixation of the capsulolabral complex on the native glenoid around the transferred graft with cinch stitches and knotless suture anchors (A), thus partially covering the graft (B).

The capsulolabral complex is reattached to the glenoid around the graft with 3 cinch stitches and 3 single-loaded 2.9-mm knotless suture anchors (PushLock; Arthrex) placed in the native glenoid, thus partially covering the graft to restore anatomy (Fig 6). Alternatively, other suture and anchor configurations can be used.

## Discussion

In the treatment of anterior shoulder instability with glenoid bone loss, free bone graft transfers have proven to be a viable anatomic alternative to the commonly performed, nonanatomic Latarjet procedure.^6^^,^^18^ In particular, implant-free fixation of the free bone grafts has rendered excellent short- and long-term results.^6^ However, a drawback remains the source of the graft. Grafts are commonly harvested from the iliac crest, with the associated risk of postoperative pain, immobility, blood loss, hematoma, and nerve injury.^8^, ^9^, ^10^ Additionally, many patients understandably would like to avoid having a procedure performed in a second body region. The alternative option, allografts, can be associated with additional costs, potentially lower graft quality and healing potential, higher osteolysis rates, and risk of disease transmission.^11^, ^12^, ^13^^,^^18^

The arthroscopic bone block cerclage technique using a tricortical scapular spine autograft provides an anatomic arthroscopic glenoid reconstruction with the combined benefit of sparing the subscapularis, metal-free fixation, and intraregional donor site for autograft harvesting. The advantages and disadvantages, as well as some pearls and pitfalls of the technique, are described in Tables 1 and 2. Although the size of the graft is limited, CT-based studies have shown that the scapular spine has dimensions similar to the coracoid, and even the iliac crest, in the majority of patients^11^ and therefore is suitable as a graft choice for bony glenoid reconstruction in anterior shoulder instability. Not only does the use of grafts with smaller size make it technically easier to transfer the graft into the joint and attach the capsulolabral complex around the graft, but it has also been shown that, most of the time, iliac crest bone grafts are too large and therefore augment rather than reconstruct the glenoid, which leads to extensive resorption due to remodeling processes based on Wolff’s law.^19^ Furthermore, in the case of failure, revision surgery with an iliac-crest bone graft transfer or Latarjet is easily possible, as the donor sites are still available, no metal implants were used, and the subscapularis was spared.

**Table 1:** Advantages and Disadvantages

Advantages
• Anatomic reconstruction
• Preserved integrity of the subscapularis tendon
• No metal implants
• Intraregional donor site
• Easy to revise
Disadvantages
• Limited graft size
• Difficult to switch to open surgery intraoperatively, as the procedure is performed in lateral decubitus position

**Table 2:** Pearls and Pitfalls

Pearls
• Horizontal posterior approach for better placement of the drill guide
• A harvest location 4 to 5 cm lateral to the medial scapular border to obtain the largest cross-sectional graft
• Expansion of the anterior interval approach using index finger for easier graft passage through the rotator interval
Pitfalls
• Drill guide malpositioning can lead to tunnel malalignment and graft malpositioning
• Acromion base fracture due to improper harvest location too far lateral on the spina scapulae
• Suture cut-through of the graft in patients with poor bone quality

## Supplementary Data

ICMJE author disclosure forms

Video 1 Arthroscopic bone block cerclage technique using a tricortical scapular spine autograft for glenoid reconstruction in patients with anterior shoulder instability.
